# Are Gender Differences Important for Autoimmune Liver Diseases?

**DOI:** 10.3390/life14040500

**Published:** 2024-04-12

**Authors:** Annarosa Floreani, Daniela Gabbia, Sara De Martin

**Affiliations:** 1 Scientific Consultant IRCCS Negrar, 37024 Verona, Italy; 2 University of Padova, 35122 Padova, Italy; 3 Department of Pharmaceutical and Pharmacological Sciences, University of Padova, 35122 Padova, Italy; daniela.gabbia@unipd.it (D.G.); sara.demartin@unipd.it (S.D.M.)

**Keywords:** gender differences, primary biliary cholangitis, primary sclerosing cholangitis, AIH, IgG4-related disease

## Abstract

Gender Medicine has had an enormous expansion over the last ten years. Autoimmune liver diseases include several conditions, i.e., autoimmune hepatitis (AIH), primary biliary cholangitis (PBC), primary sclerosing cholangitis (PSC), and conditions involving the liver or biliary tree overlapping with AIH, as well as IgG4-related disease. However, little is known about the impact of sex in the pathogenesis and natural history of these conditions. The purpose of this review is to provide an update of the gender disparities among the autoimmune liver diseases by reviewing the data published from 1999 to 2023. The epidemiology of these diseases has been changing over the last years, due to the amelioration of knowledge in their diagnosis, pathogenesis, and treatment. The clinical data collected so far support the existence of sex differences in the natural history of autoimmune liver diseases. Notably, their history could be longer than that which is now known, with problems being initiated even at a pediatric age. Moreover, gender disparity has been observed during the onset of complications related to end-stage liver disease, including cancer incidence. However, there is still an important debate among researchers about the impact of sex and the pathogenesis of these conditions. With this review, we would like to emphasize the urgency of basic science and clinical research to increase our understanding of the sex differences in autoimmune liver diseases.

## 1. Introduction

The disease burden of autoimmune liver disease includes autoimmune hepatitis (AIH), primary biliary cholangitis (PBC), primary sclerosing cholangitis (PSC), and overlapping conditions between AIH and PBC or PSC. A complete review on gender disparities in liver disease, including autoimmune liver disease, has recently been published [1]. Indeed, these disparities imply a wide contrast, not only regarding the medical point of view, but also social policies, employment, housing, health care, behavior factors, and cultural and social values [2]. In fact, the terms sex and gender are usually referred to as biological or socio-cultural issues, respectively. Actually, all the approaches often comprise both biological and social dimensions [3]. As already outlined [1], most studies describing autoimmune liver diseases have focused on one sex only and less on the comparative aspects. Men and women are differently affected by diseases in general and also by liver diseases; thus, sex-stratified medicine is an extremely important, although understudied, facet of medical care [4]. In general, clinical data suggest that men and women exhibit differences regarding the epidemiology and the progression of many diseases, including liver diseases [5,6], and there is an urgent need for basic science and clinical research to increase our understanding of gender differences in these contexts [7].

The aim of this paper is to review the current knowledge regarding the influence of sex on the setting of autoimmune liver diseases, underlining the relevance of sex-specific analysis in the prognosis and management of these diseases. To provide a state-of-the-art analysis of this topic, a review of the literature in PubMed, Scopus, and Science Direct was conducted (included dates: 1999–2023).

## 2. Autoimmune Hepatitis

Autoimmune hepatitis (AIH) is a rare inflammatory liver disease which can progress to end-stage liver disease and liver failure [8]. The point of prevalence in Northern Europe is estimated at 10–20 per 100,000 subjects, with an incidence rate of 1.2 per 100,000 person-years [9] and a 1:3.5 male–female ratio [5]. In a retrospective cohort of adults in the Optum Clinformatics Data Mart (2009–2018) in the USA, AIH incidence was 4 per 100,000 person-years and the prevalence was 26.6 per 100,000 [10]. Male sex, black race, and Hispanic ethnicity were associated with AIH with cirrhosis [10].

An exhaustive review of the pathogenesis of AIH has recently been published [11]. It has been estimated that approximately 9–12% of patients with AIH experienced a drug-induced hypersensitization process, and, of these, most were women [12]. In a preclinical in vivo study, Thomas and collaborators [13] immunized mice with an epitope of CYP2E1 and showed that IL-4 activated CD4+ T cells, thereby inducing the recruitment and influx of neutrophils, macrophages, and mast cells into the liver. They also showed that IL-33-induced FOXP3+ regulatory T cells to confer protection against drug-induced autoimmune hepatitis in female and male mice. This work also confirms the previous results implicating female sex, by demonstrating that female BALB/C mice develop a more severe drug-induced autoimmune hepatitis [14].

### 2.1. Factors Responsible for Gender Differences

**Figure 1:**
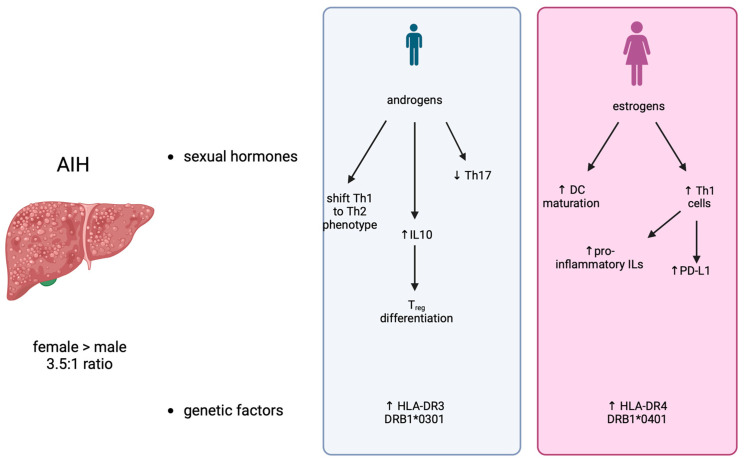
Sex dimorphism and AIH.

The most important factors responsible for gender differences in AIH include: (1) sexual hormones; (2) genetic factors; (3) environmental factors; and (4) microbiome (Figure 1).

Sexual hormones. Insight about the role of sex hormones in AIH has been obtained by observations during pregnancy. Interestingly, pregnancy in AIH patients presents an increased risk of complications, both fetal and maternal [15], with respect to healthy women, although the successful delivery of healthy newborns has been reported in most AIH patients. On the other hand, it should be noticed that pregnancy may have a significant impact on the history and outcome of AIH. For example, up to 50% of patients encounter flares in AIH activity after delivery, or flares or AIH manifestations during or immediately after pregnancy [16,17]. Furthermore, the onset of de novo AIH has also been observed after delivery [18]. On the contrary, in many AIH patients, disease severity is reduced during pregnancy, so that the therapy’s dosage can be reduced [16,19]. From the mechanistic point of view, estrogens influence immune system activity, mainly acting on dendritic cells (DC). Evidence shows that these hormones can either promote DC maturation, leading to an increase in T helper 1 (Th1) cell activity, due to the increased production of proinflammatory interleukins (ILs) [20,21,22], or they can establish a tolerogenic immune environment by upregulating the expression of checkpoint inhibitors, such as PD-L1 [23,24]. On the other hand, androgens may also influence the pathogenesis of AIH by shifting the Th-1 to the Th-2 phenotype [25], thus reducing Th-17 cells in the liver [26] and increasing the secretion of IL-10, which drives the differentiation of regulatory T cells (Tregs) [27]. Notably, human and mouse T cells express cytosolic androgens and membrane-bound androgen receptors (mAR); androgens led to changes in cytokine expression in T cells either directly or indirectly via antigen presenting cells, with a shift toward a decrease in pro-inflammatory cytokine expression [28].

Genetic factors. Genes related to immune system functions are encoded on the sexual X and Y chromosomes [29]. AIH has been significantly associated with human histocompatibility complex (HLA) haplotypes. The most significant associations emerged in the alleles encoding for HLA-DR3 and DR4 [30]. A sex dimorphism was evident, since male AIH patients were characterized by an increased expression of HLA-DR3 (DRB1*0301) [31], whereas HLA-DR4 (DRB1*0401) was increased in female patients [32]. Furthermore, DRB1*0301 patients show an earlier onset of AIH, which also has more severe biochemical and histological features than those observed in the other haplotypes, as well as worse outcomes [32,33]. On the other hand, DRB1*0401 patients often have other immunological comorbidities but also higher response rates to the pharmacological treatments [32,33].

Environmental factors. Environmental factors, such as chemicals or viruses, can represent the triggers for AIH development [34]. Environmental factors can lead to the initialization of the autoimmune response by four different mechanisms. (1) The massive non-specific activation of resting T cells, which can be exerted either by viral infections, such as, for example, the Epstein–Barr virus (EBV) [35], or xenobiotics, like Concavalin A, is able to induce the secretion of proinflammatory cytokines [36]. (2) The creation of a neoantigen by the binding of a self-protein with a chemical compound, which has been reported both for natural products, such as black cohosh [37], and drugs, like statins [38]. (3) Molecular mimicry, i.e., the cross-reactivity between a viral and a self-protein, which has been proposed for HCV and SARS-CoV2 patients [39]. (4) The modulation of gene expression, exerted by molecules triggering an autoimmune reaction due to the creation of a pro-inflammatory microenvironment, ideal for autoantigen presentation [34].

Microbiome. It is well known that dysbiosis, i.e., the pathological changes of the gut microbiome, plays a pivotal role in driving aberrant immune responses and is fundamental in the development of autoimmune diseases [40], including AIH [41], although no information about the possible effect of sex is available so far for these patients. However, it is well known that AIH patients are characterized by a significant reduction in gut microbiome diversity, with increased relative abundance of aerobic or partially anaerobic microorganisms [42]. Mechanistic findings obtained in animal models demonstrated that the dysregulated interaction between the gut microbiome and mucosal immune system might be pivotal in AIH pathogenesis, but the molecular mechanisms linking AIH development and the microbiome needs to be further investigated [43]. Recent studies have confirmed that processes driven by the gut microbiome, including the alteration of gut permeability, the migration of gut microbes or their byproducts, and the disruption of immune homeostasis, are main actors in AIH development and progression [44,45]. In this context, the modulation of the microbiome can be exploited as a therapeutic target. For example, the genus_Clostridium_innocuum_group has recently been identified as protective against AIH [46], suggesting a possible role for microbiome modulation in the therapy of this disease, and autoimmune liver disease in general.

### 2.2. Clinical Course

Male gender was identified as a risk factor for adverse outcomes, including the development of hepatocellular carcinoma (HCC) [9]. Furthermore, a nationwide study of Danish patients with AIH showed that mortality was increased in patients with multiple extra-hepatic diseases [47].

The impact of gender and race on the outcome of patients with AIH has been analyzed using a National Hospital Registry in the USA [48]. Using the propensity score method, a total of 9218 patients were analyzed, of which 4609 were females and 4609 were males. For the race comparison, 3688 African American patients and 3173 Hispanics were analyzed, with an equal number of whites. In multivariate analysis, females were less likely to develop complications of portal hypertension or acute liver failure (ALF). When comparing races, black patients had higher rates of ALF and hepato-renal syndrome, but lower rates of encephalopathy. Hispanics also exhibited higher rates of hepatic complications, including ascites, variceal bleeding, spontaneous bacterial peritonitis, and encephalopathy.

Patients with AIH have been shown to carry a 1.5 times higher 10-year risk of cancer than matched controls from the general population [49]. The absolute 10-year risk of any cancer was highest in the patients with cirrhosis and in those with older age. A recent meta-analysis of 39 studies showed that the overall incidence of HCC was 3.53 per 1000 person-years [50]. The risk of HCC was significantly higher among males that among females, with an over two-fold higher risk as a whole. Further analyses revealed that the risk of HCC was also correlated with the presence of cirrhosis, especially in Asian populations [48,49,50].

It is not known whether a gender disparity exists in terms of response to immunosuppressive treatment, but it is well known that many factors, including sex, age [51], and liver dysfunction [52], can influence the pharmacokinetics and pharmacodynamics of drugs. However, about 10% of patients with AIH present normal levels of gamma-globulins. Those patients are indistinguishable from patients with typical AIH, based on the prevalence of concomitant autoimmune conditions, histological changes, laboratory markers, and response to treatment. It has been hypothesized that this subgroup has a higher chance of drug withdrawal [53].

## 3. Primary Biliary Cholangitis

Primary biliary cholangitis (PBC) is a chronic autoimmune liver disease characterized by immune-mediated destruction of small and medium intrahepatic bile ducts that lead to cholestasis [54] and eventually progresses to cirrhosis and liver failure [55,56], characterized by a complex and not completely defined pathogenetic mechanism [57]. The incidence and prevalence of PBC differ according to region, with increased prevalence in Northern Europe [58]. In the United States, the prevalence of PBC has been shown to be variable according to age, sex, and race, as demonstrated in a study from the Fibrotic Liver Disease Consortium that reports the highest prevalence in white women aged 60–70 years [59]. The predominant population affected by PBC are middle-aged women, with an estimated 1 in 1000 women over the age of 40 affected globally [60].

The specific female preponderance is well known [61]. The F/M ratio of 9:1 has been described in several series of patients; however, more recently, more incident cases of males with PBC have been observed. Unless most of the recent studies have been performed with administrative data, the F/M ratio tends to be lower than previously reported.

### 3.1. Factors Responsible for Gender Differences

**Figure 2:**
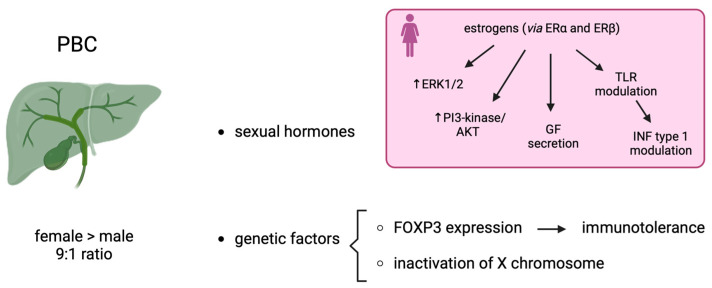
Sex dimorphism and PBC.

Factors responsible for gender differences in PBC include sexual hormones and genetic factors (Figure 2).

*Sexual hormones.* Estrogen receptor alpha (ERa), which may play a significant role in cholestasis, is highly expressed in PBC [62]. However, both ERa and ERb modulate the response of cholangiocyte to damage activating intracellular cascades involving ERK1/2 (extracellular regulated kinase 1/2) and PI3-kinase/AKT (phosphatidylinositol-3′kinase), which are involved in the signaling pathways of growth factors, such as nerve growth factor and endothelial factor. Moreover, estrogen stimulates the secretion of different growth factors related to the proliferation of cholangiopathies [63].

Furthermore, high levels of estrogens drive the immune response from the Th1 to the Th2 phenotype [64]. T helper cells are critical for the progression of PBC. This has been demonstrated in a mouse model of autoimmune cholangitis, which has a notably female predominance [65]. Mechanistically, the knockout of interferon type I signaling prevents this female-prevalent autoimmune cholangitis phenotype [66]. Although an effect directly exerted by estrogens on IFN-γ expression has not yet been demonstrated, it is well known that sexual hormones can modify the expression of pattern of recognition receptors, such as Toll-like receptors (TLR) [67]. Therefore, this modulation can have an impact on the levels of type 1 IFN, indirectly. Furthermore, preclinical in vivo data indicate that estrogens can have, per se, a homeostatic positive effect on cholangiocytes [3]. This observation is corroborated by the loss of ER expression in patients with PBC of a severe grade [68]. Little is known about the effect of testosterone on the growth and survival of cholangiocytes. In male rats, androgen receptors (AR) are expressed by cholangiocytes, and testosterone stimulates their proliferation in a rodent model of cholestasis [69].

*Genetic factors.* An important issue for understating the higher prevalence of PBC in females was the inactivation of the X chromosome [70]. During embryonic development, one of the two X chromosomes is randomly inactivated in females; this process, under normal conditions, results in a cellular mosaicism where about one-half of the cells in a given tissue express either the maternal X or the paternal chromosome. However, since X chromosome inactivation is not complete, with 15–23% of genes escaping inactivation, a female-specific heterogeneous population of cells with biallelic expression of some X-linked genes is emerging. A recent elegant chromosome X-wide association study analyzed 5.244 case patients with PBC and 11,875 controls [71]. Indeed, a significant locus has been identified; this locus is characterized by a super-enhancer targeting all the genes of the region, including FOXP3. FOXP3 is a specific marker of Tregs, which are critical for the maintenance of immune tolerance [72]. Moreover, FOXP3 RNA expression levels in the whole blood show a significant differential expression between males and females.

### 3.2. Clinical Course

The largest cohort of hospitalized patients with PBC (between 2007 and 2014) was recently published in the USA using the National Inpatient Sample records [73]. The median age was 62 ± 14.8 years, and the ample was made up of 81% females. Males were younger than females (58.6 vs. 62.7 years). The clinical features of chronic liver disease were similar in both genders.

A retrospective study conducted in Taiwan on 75 PBC patients [74] showed that 84% of cases were females with a median age of 64.6 ± 1.78 years, whereas males had a median age of 46.6 ± 5.6 years. Male patients showed fewer extrahepatic autoimmune disorders and more severe liver injuries than females. Interestingly, this study analyzed the relationship between androgen response expression and systemic inflammation using the blood transcriptome of 90 PBC patients. The gene set variation analysis (FSVA) showed a positive significant correlation between peripheral mononuclear cells (PMNCs), the androgen receptor response, and the inflammatory response. It can be argued that this factor can explain the more severe course of PBC in males than in females, but more data are needed to further explain this hypothesis.

In PBC there is also a strong association between male gender, advanced disease, lack of response to ursodeoxycholic acid (UDCA), and the risk of HCC [75,76,77]. Moreover, it is known that the incidence and mortality rates of HCC are 2–5 times higher among men than women [78]. It has been hypothesized that the sex disparity between males and females is due to the effect of estrogens that protect hepatocytes from malignant transformation throughout the downregulation of Interleukin 6 (IL-6) and its release from Kupffer cells [79]. Although the modulation of the microbiome has been suggested as a possible therapeutic target [80], the role of sex in this context still needs to be studied and understood.

## 4. Primary Sclerosing Cholangitis

Primary sclerosing cholangitis (PSC) is a rare disease characterized by progressive cholestasis and by intrahepatic and extrahepatic fibro-inflammatory biliary strictures [81,82]. PSC mainly affects relatively young males and is strongly associated with inflammatory bowel disease (IBD). The prevalence of IBD in a recent systematic meta-analysis was 71.1%, most commonly found in ulcerative colitis [40]. On the contrary, a systematic meta-analysis, including 64 studies for a total of 776,700 patients, showed that the pooled prevalence of PSC in IBD was 2.16% [83]. The prevalence was higher in men with IBD (2.09%) compared with women (1.79%), *p* < 0.001. Overall, the highest pooled prevalence was observed in South America and the lowest in South East Asia [84].

Different phenotype variants can be recognized: classic large-duct PBC characterized by multifocal intra- and extrahepatic strictures with ductal dilatation with or without association with IBD, small duct PSC characterized by typical histological changes in absence of radiographic abnormalities and overlap syndrome with AIH and PSC associated with elevated levels of IgG4. In PSC, the male–female ratio is 2:1. A systemic review [85], including eight studies from North America and European countries, estimated a PSC incidence rate of 0.77 per 100,000 person-years. However, when excluding the two non-population-based studies, the incidence rate increased to 1.00 [85]. Similar rates were reported by Boonstra et al. in a systematic review of 11 studies from North America and Europe [58]. Furthermore, a recent systematic review of population-based studies reported the highest incidence in Northern Europe (Finland, 1.58, and Norway, 1.3, per 100,000 subjects, respectively) and Minnesota (1.47), with the lowest rate observed in the Mediterranean area (Italy, 0.1) [86]. Using the database from the Italian Epidemiological Rare Disease Registry, the crude annual incidence of PSC in females increased from 0.05 per 100,000 in 2012 to 0.09 per 100,000 in 2014, reaching a rate similar to that of males [67].

### Role of Gender in PSC

Aside from the detailed reports on the epidemiology, the role of gender and sex hormones in PSC is virtually ignored [87]. However, an international study, which included 7121 patients with PSC from Europe, North America, and Australia, showed that female sex was associated with a lower risk of liver transplant or death and with a lower risk of hepatobiliary malignancies [88]. Moreover, the I148M variant polymorphism was investigated in two different cohorts of PSC: a German cohort (including 121 prospective subjects) and in a Norwegian cohort with 347 subjects (234 of whom received ERC and 123 of whom had a dominant stenosis) [89]. The results showed that male carriers of the I148M variant showed significantly reduced actuarial survival free of liver transplantation (*p* = 0.013) compared to male wildtype patients. Again, the I148M polymorphism did not affect female PSC patients with bile duct stenosis.

**Figure 3:**
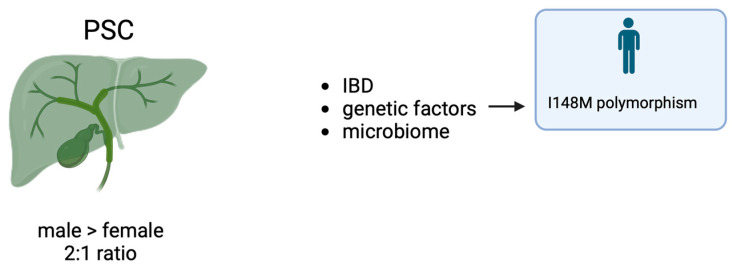
Sex dimorphism and PSC.

Microbiome. RNA sequencing studies demonstrated that PSC patients have an altered gut microbiome [90,91,92]. In particular, the gut microbiome in PSC patients is significantly different than that of healthy controls, being characterized by a drop of microbial diversity and differences in the amounts of specific bacteria, especially Enterococcus and Veillonella [90,91]. The interesting study by Rühlemann and collaborators pointed out that the peculiar and reproducible microbiota composition of PSC patients is modelled by the disease itself and not influenced by environmental factors [90] or inflammatory bowel disease (IBD), which is frequently present in PSC patients and vice versa [91]. Since Enterococcus positively correlates with ALP levels, it has been suggested that a link might exist between these bacteria and PSC severity [91] (Figure 3).

The risk factors for AIH, PBC, and PSC displaying sex dimorphism are reported in Table 1.

## 5. Overlap Syndromes

Overlap syndromes between AIH and PBC or PSC have been recognized since the 1980s. However, the concept of overlapping has been changed over the years. As far as PBC is concerned, the term AIH/PBC overlap syndrome has been considered as the coexistence of the two separate diseases with a variable onset during their natural history: (1) development of cholestatic changes during the course of AIH; (2) development of autoimmune features during the course of PBC; (3) simultaneous diagnosis of AIH and PBC. The diagnostic approach of this variant has been generally adopted according to the “Paris criteria” based on the simultaneous or consecutive presence of at least two out of three biochemical, serological, and histological criteria for both PBC and AIH [93]. However, the diagnostic criteria imply a morphologic approach through liver biopsy, thus unanswered questions remain, including the correlation between the clinical aspects and the morphology [94]. In a systematic review, which included 17 studies of PBC/AIH comprising a total of 402 patients, female gender was present in 87–100% of either retrospective or prospective studies [95].

The AIH/PSC overlap syndrome leads to some interesting observations. This condition has been described in both children and adults. In a large pediatric cohort, which included 781 subjects with PSC, 33% of cases had an overlap syndrome with AIH [96]. Patients with overlap were more likely to be female and more likely to have an association with IBD. Moreover, age, gender, and AIH overlap did not impact long-term outcome.

In childhood, the term “autoimmune sclerosing cholangitis” was preferred to AIH/PSC overlap syndrome [97]. However, quite recently, a new concept has arisen based on studies on the natural history of PSC and its variants. As hypothesized [98], autoimmune sclerosing cholangitis and PSC/AIH overlap syndrome represent temporal phases along a fundamental PSC continuum. In this view, there is a need for implementing prospective studies on clinical trials involving immunosuppressant and new molecules, and prospective studies on the natural history of PSC as well.

## 6. IgG4-Related Disease

IgG4-related disease (IgG4-RD) potentially involves all organs of the body, presenting with mass forming lesions and characterized by IgG4-positive plasma cells infiltrating the tissues [99]. A Chinese study evaluated the sex disparities in clinical characteristics and prognoses of IgG4-RD [100]. Four hundred and three patients (150 females and 253 males) were included, with a male–female ratio of 1.69. Male sex was significantly predominant compared to females; the significance was more pronounced in male patients older than 60 years. This point is particularly important, because in a previous report, which included a group of 25 patients ≤17 years of age, a slight female predominance was found [101]. In general, female patients presented Mikulicz’s disease and thyroiditis more frequently, while, in male patients, autoimmune pancreatitis, sclerosing cholangitis, and retroperitoneal fibrosis were more frequent. Moreover, in the group treated with steroids, male sex was independently associated with a worse prognosis.

In another multicenter study, which included 184 Latin Americans with IgG4-RD, men and women were equally affected by this condition [102]. However, male sex was significantly associated with the biliary tract phenotype with kidney and retroperitoneal involvement.

IgG4-related cholangitis (IgG4-RC) is the major hepatobiliary manifestation of IgG4-RD and involves mainly men with a median age of >55 years [103]. It is noteworthy, however, that the clinical presentation of IgG4-RC often mimics other biliary diseases, such as PSC and cholangiocarcinoma [103].

## 7. Sex Disparities in Autoimmune Liver Diseases in Pediatrics

A comprehensive review of juvenile AIH has recently been published [104]. Sixty to eighty percent of patients with AIH are females, and 40% have a family history of autoimmune disorders. In general, children with AIH, similarly to adults, have the same prevalence of female sex, as summarized in Table 2.

It should be stressed that mortality is still present, reaching 28.5% in Ghana [105].

More recently, a 30-year follow-up of 159 patients with childhood onset autoimmune liver disease has been published [112]. One hundred and nineteen children presented with AIH. Female gender was predominant in both type 1 AIH (63%) and type 2 AIH (67%). Interestingly, during follow-up, the following biliary disease progression was observed: 19.8% of patients with type1 AIH developed biliary features by adulthood and 50% of them developed a classical PSC [112].

Moreover, a cohort of 117 children diagnosed with AIH between 1973 and 2002 underwent a median follow-up of 20 years [113]. Type 2 AIH showed a higher prevalence of females than the group with type 1 AIH (45/52 vs. 41/65, *p* = 0058). A sustained remission after treatment withdrawal was recorded in 24%, and, in terms of liver outcome, there were no differences between type 1 and type 2 AIH.

PBC is virtually absent in childhood. There are only anecdotal cases so far described in the literature of pediatric-onset PBC. The first report described two cases of PBC diagnosed at 16 and 15 years of age in 2 girls in Canada [114]. The third case reported in the world was seen in a 17-year-old young woman who suffered from a previous infection of *Borrelia burgdorferi* [115]. One further case of type 2 AIH associated with an unexpected and transient presence of PBC-specific AMA has been described by Invernizzi et al. [116]. Finally, the youngest case of PBC has been described in a 5-year-old female child presented with jaundice and encephalopathy [117]. All cases described in the literature of PBC-onset in childhood are females.

There is a very high number of reports on PSC in childhood. A review by Giorgina Mieli-Vergani and Diego Vergani focused on the unique features of PSC in children [118]. Young patients with PSC frequently displayed features of autoimmunity (one third of whom having AIH/PSC overlap syndrome). Moreover, they experienced a beneficial effect from immunosuppressive therapy, which is in contrast with adults who have disappointing effects with such treatment. In another review, the same authors observed that the mode of presentation of autoimmune cholangitis was similar to type 1 AIH, but the bile duct disease progressed in half of them, leading to liver transplant [119]. Furthermore, the authors observed that cholangiocarcinoma rarely develops in children compared to adults, in whom it is a frequent complication of PSC [119].

Indeed, it has been stressed that PSC is rare in pediatrics, whereas sclerosing cholangitis is associated with strong autoimmune features [120]. In a retrospective cohort from the King’s College Hospital, which included 83 children with autoimmune sclerosing cholangitis, female sex accounted for 42% of cases, while in a prospective cohort, which included 27 patients, female sex was present in 55% of cases [97]. According to the more relevant studies on juvenile sclerosing cholangitis, the prevalence of males ranged between 57% and 65% [97]. Unfortunately, none of the reports on autoimmune sclerosing cholangitis in pediatrics focuses on gender disparities in clinical features or outcomes. Prospective studies on large cohorts of children with sclerosing cholangitis are needed in order to better understand gender disparities in this condition. Moreover, many open questions need to be addressed with the collaboration of many experts in the field of both pediatrics and adults [121].

## 8. Conclusions and Future Perspectives

Factors contributing to sex-related and gender-related modulation of autoimmune liver diseases and their outcomes in women and men are extremely important. Sex-related factors, including genetic, epigenetic, sex hormones, and gene–hormone interaction, are not fully understood. The best paradigm is either AIH or PBC. Genetic factors, similarly to other complex diseases, are not linked to a specific gene; HLA-linked genes are important for disease susceptibility. Concerning AIH, in Northern Central America, an association with DRB1*0301 and DRB1*0401 alleles has been described, while in Brazilians, DRB1*13 and DRB1*03 seem to be the more prevalent [122]. However, associations with single nucleotide polymorphisms within HLA and non-HLA genes have been assessed in autoimmune liver diseases but do not seem to explain gender disparities.

The interaction between genes and sexual hormones do represent a clue factor in the pathogenesis of these diseases. Estrogens and their fluctuations during the reproductive and non-reproductive life may modulate the course of the disease. It is well known that AIH is less invasive in pregnancy, and, during this phase, there is a reduced need for immunosuppressive therapy. In the case of PBC, its early onset in a young woman causes a more progressive liver disease with higher risk of complications. On the other hand, a late onset in a post-menopause woman frequently leads to a mild disease. PBC in men is rather infrequent, although more cases are being diagnosed in recent years. A man with PBC has a higher risk of developing HCC, mainly in cases of advanced disease or if they do not respond to UDCA. This observation implies a different strategy for optimizing the follow-up of the disease. Female patients with mild disease can undergo a physical examination every 8–12 months, with an ultrasound every year. Female patients with advanced disease should have a visit every six months and an ultrasound every six months as well. Male patients with PBC should have a regular follow-up every six months, optimizing a program for the prevention of HCC every six months.

In regard to PSC, although male sex is more frequent, the follow-up strategies should be the same in both sexes, because of the double neoplastic risk: cholangiocarcinoma and colorectal cancer for those with associated IBD.

Future directions for research mainly concern the eventual gender disparities in responding to treatment and the consequent need to well differentiate the response to treatment according to gender in clinical trials. More urgent perspectives regarding the importance of gender disparities among autoimmune liver diseases are included within the concept of “rare disease”, according to the Reference Network for Hepatological Disease (ERN RARE LIVER). It is well known that AIH, PBC, and PSC are classified within the regulation for ERN RARE LIVER in Europe, whereas in Italy only PSC is considered a rare disease. New expectations for autoimmune liver diseases concern a dramatic change in their epidemiology, due to an increase in new diagnoses all over the world. Thus, it could be speculated that, in a few years, these diseases will no longer fall within the group of rare diseases. However, among the disease groups, male patients with PBC will continue to be regarded as a subgroup which needs special attention and timely referral to a specialist. Moreover, future interventions for PSC patients will be planned in both sexes for improving symptom control, preventing a terminal stage of liver disease, and preventing neoplastic complications. Finally, male sex is an important risk factor in all autoimmune liver diseases for disease progression and development of HCC. Therefore, in these cases, liver transplant is particularly important for survival.

## Figures and Tables

**Table 1:** Summary of the sex-dimorphic risk factors for AIH, PBC, and PSC.

Autoimmune Disease	Risk Factors	Results	Refs.
AIH	Increased IL-4 activated CD4+ T cells	Female BALB/C mice develop a more severe drug-induced AIH Tregs confer protection against drug-induced autoimmune hepatitis	[13,14]
	Estrogens	Estrogens promote DC maturation, leading to an increase in Th1 cells activity, increased production of proinflammatory ILs, Estrogens upregulate the expression of checkpoint inhibitors, e.g., PD-L1	[20,21,22,23,24]
	Androgens	Androgens may influence the pathogenesis of AIH by shifting the Th-1 to the Th-2 phenotype, reducing hepatic Th-17 and increasing IL-10 secretion, which drives Treg differentiation	[25,26,27,28]
	Pregnancy	Pregnancy increases risk of fetal and maternal complications and flares in AIH activity after delivery	[15,16,17]
	HLA-DR3	Increased expression of HLA-DR3 (DRB1*0301) in male AIH patients	[31]
	HLA-DR4	Increased HLA-DR4 (DRB1*0401) in female AIH patients	[32]
PBC	Estrogens	ERα and Erβ modulate cholangiocyte response to damage, activating intracellular cascades involving ERK1/2 and PI3-kinase/AKT Estrogens stimulate the secretion of different growth factors in proliferating cholangiopathies and shift Th cells from Th1 to the Th2 phenotype Sexual hormone regulation of TLRs affect type 1 IFN that may impact on PBC progression Estrogens exert a homeostatic positive effect on cholangiocytes	[3,62,63,64,67]
	Androgens	Testosterone stimulates AR proliferation cholangiocytes in a rodent model of cholestasis	[69]
	Inactivation of X chromosome	Female-specific heterogeneous population of cells with biallelic expression of some X-linked genes with a locus in which there is a super-enhancer targeting genes, e.g., FOXP3, affect Tregs and are critical for the maintenance of immune tolerance	[71,72]
PSC	I148M variant polymorphism	Male carriers of the I148M variant showed significant reduced actuarial survival free of liver transplantation data	[89]

**Table 2:** Epidemiology of AIH in recent cohorts of children.

Country (Ref.)	Type	N.	Female %	Acute Onset	Age (Mean or Median Years)	Mortality
Ghana [105]	Retrospective	13	61.5%	15.7%	10 (5–13)	28.5%
Iran [106]	Retrospective	86	66.27%	-	9.1 ± 4.36	10.5%
Saudi Arabia [107]	Cross-sectional	25	56%	16%	9.4 ± 4.2	0%
Egypt [108]	Retrospective	34	64.7%	51%	8 ± 3	2.9%
Scotland [109]	Prospective	30	64%	44.4%	11.4 (1–15.9)	0%
Switzerland [110]	Retro- and prospective	30	53%	-	12.5 (8–15)	0%
Jordania [111]	Retrospective	16	75%	31.3%	9.4 ± 4.13	18.8%

## Data Availability

Not applicable.
